# Challenges and recommendations for measuring time devoted to implementation and intervention activities in health equity-focused, resource-constrained settings: a qualitative analysis

**DOI:** 10.1186/s43058-023-00491-7

**Published:** 2023-09-01

**Authors:** Douglas E. Levy, Deepinder Singh, Kelly A. Aschbrenner, Madeline E. Davies, Leslie Pelton-Cairns, Gina R. Kruse

**Affiliations:** 1 Mongan Institute Health Policy Research Center, Massachusetts General Hospital, 100 Cambridge St., Suite 1600, Boston, MA 02114 USA; 2 Harvard Medical School, Boston, MA 02115 USA; 3 Kraft Center for Community Health, Massachusetts General Hospital, 125 Nashua St, Boston, MA 02114 USA; 4 The Dartmouth Institute for Health Policy and Clinical Practice, Dartmouth College, Lebanon, NH USA; 5 Department of Psychiatry, Geisel School of Medicine at Dartmouth, 46 Centerra Parkway, Lebanon, NH 03766 USA; 6 Massachusetts League of Community Health Centers, 40 Court St, Boston, MA 02108 USA; 7 Division of General Internal Medicine, Massachusetts General Hospital, 100 Cambridge St., Suite 1600, Boston, MA 02114 USA

**Keywords:** Time use, Cost, Measurement, Implementation, Equity

## Abstract

**Background:**

There is little guidance for conducting health equity-focused economic evaluations of evidence-based practices in resource-constrained settings, particularly with respect to staff time use. Investigators must balance the need for low-touch, non-disruptive cost data collection with the need for data on providing services to priority subpopulations.

**Methods:**

This investigation took place within a pilot study examining the implementation of a bundled screening intervention combining screening for social determinants of health and colorectal cancer at four federally qualified health centers (FQHCs) in the Boston metropolitan area. Methods for collecting data on personnel costs for implementation and intervention activities, including passive (automatic) and active (non-automatic, requiring staff time and effort) data collection, as well as three alternate wordings for self-reporting time-use, were evaluated qualitatively using data collected through interviews with FQHC staff (including clinicians, population health staff, and community health workers) and assessments of data completeness.

**Results:**

Passive data collection methods were simple to execute and resulted in no missing data, but missed implementation and intervention activities that took place outside planned meetings. Active cost data collection using spreadsheets was simple for users when applied to care processes already tracked in this fashion and yielded accurate time use data. However, for tasks where this was not typical, and when tasks were broken up over multiple sessions, spreadsheets were more challenging to use. Questions asking about time use for a typical rather than specific time period, and for typical patients, yielded the most reliable and actionable data. Still, even the best-performing question had substantial variability in time use estimates. Participants noted that patient characteristics of interest for equity-focused research, including language spoken, adverse social determinants of health, and issues related to poverty or mental health, all contributed significantly to this variability.

**Conclusions:**

Passively collected time use data are the least burdensome and should be pursued in research efforts when possible, but should be accompanied by qualitative assessments to ensure the data are an accurate reflection of effort. When workflows are already tracked by active data collection, these are also strong data collection methods. Self-reported time use will be most accurate when questions inquire about “typical” tasks and specific types of patients.

Contributions to the literature

- Whether for research or operations, studying the implementation of evidence-based programs focusing on health equity often requires detailed economic evaluations, including assessments of time use, but these are challenging to conduct in resource-constrained settings serving priority populations.
- In these settings, there is a tension between using low-cost, low-burden data collection methods and the need for detailed data on time use for providing services to priority populations.
- We present a qualitative assessment of cost data collection methods tested in federally qualified health centers.
- We recommend passive (automatic) data collection when possible and provide additional guidance on self-reported time use when appropriate.

## Background

Costs and resource needs are consistently noted as key considerations in implementation science determinant (EPIS and CFIR) and evaluation (RE-AIM) frameworks [1–3]. Costs are also central elements of planning and implementation decisions outside the research context. This is particularly true in resource-constrained settings focused on promoting health equity, including safety net institutions such as federally qualified health centers (FQHCs) which have relatively limited flexibility in obtaining and allocating resources when implementing evidence-based practices (EBPs) [4].

Personnel time use is a challenging aspect of cost measurement [5]. Unlike opportunity costs and practice revenue impacts, which can be calculated with retrospective data, time use is best measured contemporaneously. In FQHCs, collecting data on time use, whether for research or operations planning, poses two competing methodological challenges. Having dedicated personnel to measure time use is recommended, but it is often untenable given that data collection at FQHCs should use low-burden methods, minimal FQHC staff time, and avoid disruptions to operations [6]. At the same time, it is FQHCs’ mission to provide health services in underserved areas and thus promote health equity [7]. Therefore, time use data collection must have sufficient detail to allow stratification by key subgroups, which is resource-intensive. With adequately nuanced data, economic evaluations can guide resource-constrained institutions to efficiently allocate their scarce resources while promoting health equity.

Currently, there is little practical guidance resource-constrained institutions can consult for unobtrusively collecting high-quality, subgroup-specific time use data allowing rigorous, equity-focused program evaluation and planning. The objective of our study was to conduct a qualitative assessment of the feasibility, strengths, and weaknesses of light-touch, equity-focused methods for collecting data on personnel time devoted to implementation and intervention activities in FQHC settings.

## Methods

### Setting

The current pilot study was embedded within a separate pilot hybrid effectiveness-implementation trial testing a bundled screening intervention combining colorectal cancer (CRC) and social determinants of health (SDOH; i.e., food insecurity, housing instability, or transportation needs) screening. The research took place at four FQHCs serving racially, ethnically, and linguistically diverse populations in eastern Massachusetts between September 2020 and December 2021. Sites received implementation support to monitor internal clinic data, identify gaps in outreach for subgroups of interest (as determined by the sites), and conduct rapid adaptation of the implementation strategies [8]. Data on time use for implementation and intervention delivery were collected at each site, as described below. Clinical effectiveness and general implementation outcomes are the subjects of separate reports. The Harvard Longwood Campus IRB approved the study procedures (Protocol IRB20-1232). The Dana Farber and Mass General Brigham IRBs ceded review using the SMART IRB.

### Implementation and intervention activities

To institute the bundled screening program at the FQHCs, the research staff convened site leadership (e.g., chief medical officer, population health manager, grants manager) to develop implementation strategies. The center staff, including quality improvement officers, community health workers, and clinical staff (e.g., nursing, advance practice providers, physicians, and medical assistants) then developed the workflows for creating and reviewing patient registries and delivering the bundled intervention. Each site developed a unique strategy starting with core implementation elements dictated by the research staff, then adding site-driven adaptations, with equity goals tailored to groups they identified through analytics as underserved in their particular context, for example, groups characterized by race, language, or age. Each site staffed the effort based on local quality improvement practices and technologies (e.g., use of registries and reports) and available personnel (e.g., nurses, medical assistants, community health workers) [9, 10].

### Measuring time use

In all instances, we sought to employ the least disruptive method possible for obtaining data on personnel time use.

#### Passive data collection

For implementation activities (strategic planning, workflow design), we estimated time use using passive (i.e., automatic) data collection, obtaining data from administrative records of meeting frequency, duration, and attendance during the pilot trial period.

#### Active data collection

For intervention activities, we used active (i.e., non-automatic) data collection, testing several methods. In methods 1a and 1b, on two to four occasions, the implementation support team (GK, DS, MD) asked FQHC personnel to estimate the time they devoted to bundled screening activities (record keeping, patient outreach, patient counseling) over a specified period (a week or a half-day session). In method 2, the team queried FQHC personnel on the time it took to screen a typical patient, as well as the factors that drive screening time variability (Table 1). In each case, data were obtained by the study staff using structured guides during implementation support meetings, capturing responses using audio recordings and detailed notes. The specific FQHC personnel surveyed varied by site according to their individual implementation strategies and included quality improvement leads, nurse practitioners, navigators/community health workers, project managers, medical assistants, population health managers, and lab technicians. In method 3, FQHC personnel tracked their bundled screening efforts in real-time using spreadsheets, noting the time devoted to specific activities (outreach, counseling, etc.) on a patient-by-patient basis.

**Table 1:** Observations regarding staff responses to alternate active time-use data collection methods

Question	Observations regarding staff response
*Method 1a*	
Survey item: “I’d like you to think about your experience with dual screening over the past week. By dual screening, we mean screening for both social determinants of health and arranging FIT testing in the same outreach session. To the best of your recollection, how many hours did you spend on dual screening?”	• Staff at three of four sites (sites B, C, D) provided specific estimates of time spent on dual screening in a given week. Times varied week to week depending on competing priorities • At one of the four sites (site A), the staff reported it was difficult to say how much time they spent on bundled screening in the past week *because* of the competing priorities
*Method 1b*	
Survey item: “Thinking about the past month, in a typical half-day session, how much time do you spend managing colorectal cancer and social determinants screening for your patients, including documentation, time in clinical encounters, and time coordinating care?”	• Staff at three of four sites (sites A, B, C) responded with the number of patients contacted about bundled screening in a half-day session rather than indicating how much *time* was devoted to outreach about bundled screening in a typical half-day session • On one occasion, the staff at one site (site A) replied with a time estimate • Staff at one site (site D) indicated they could not make an estimate
*Method 2*	
Survey item: “How much time do you think you spend on these activities for a typical patient?”	• Staff at three of four sites (sites A, B, D) provided time ranges. The ratios of the longest to shortest times in the ranges varied from 1.3 to 5 • Staff at one site (site C) did not provide time estimates per patient
Survey item: “Are there some patients that take significantly longer than others? If so, how much longer and why?”	• Staff across sites noted patients with the following characteristics take longer: o Need an interpreter o Patient has many questions o Incorrect contact information o Positive screen for adverse SDOH o Mental health or cognitive issues • Staff did not ultimately estimate the extra time required for these patients
*Method 3*	
Spreadsheet tracking	• Each site used spreadsheets for some time use tracking • When collected, time-use data had plausibly narrow ranges with occasional outliers • Existing spreadsheets not set up to track the time use of follow-up calls

Survey respondent roles: population health manager, nurse practitioner, navigator, medical assistant, and lab supervisor

### Evaluation of time use measurement strategies

For passive data collection, we assessed the strengths and weaknesses of the data collection methods by asking the FQHC staff to reflect on whether all relevant implementation activities were accurately captured through administrative records. For active data collection, the strengths and weaknesses of each method were evaluated by assessing the degree to which (1) respondents were able to answer the questions at all, (2) respondents provided answers directly addressing the questions posed, and (3) the responses provided data with a degree of specificity (i.e., narrow range) that would allow cost estimates with acceptable uncertainty.

Further assessments of the feasibility, strengths, and weaknesses of these time use data collection tools were discussed as a secondary aim within focus groups conducted with the FQHC staff for the adaptation study. A 1-h focus group was conducted by an experienced facilitator (KA) over a video conference at each site using a semi-structured interview guide. Questions on time use data collection are presented in Table 2. Participants were those staff involved directly in the bundled screening intervention, either as personnel conducting the screenings or as supervisors or support staff. Each session was recorded and transcribed. From these data, the lead author (DL) identified emergent codes. These were then reviewed and adjudicated by members of the study team (GK, KA), yielding salient themes and quotes [11]. Comportment with the “Standards for Reporting Qualitative Research” guidelines is noted in the online supplement [12]. In addition to the focus groups, at the close of the time use data collection, the FQHC staff were asked to review the recorded data and identify any additional implementation or intervention costs that were not captured.

**Table 2:** Time use-related questions in focus group interview guide

Question	Exemplar quotes
If your health center wanted you to measure the effort/time you are spending on a particular task to understand the time burden, what would be your ideal method for doing that?	• “…So the actual calls are pretty easy to track. So we can just be like, ‘Okay. I make calls from this time to this time. And this is how many calls that we made.’ So that’s pretty easy to track. We can either put it on the calendar or put it into an Excel sheet.” (Site B) • “…We felt that we since we are tracking through a spreadsheet, we’re going to just create a column so that when they call patients, every time– the amount of time that they spend, they just document it as part of the process. So they felt that was one of the easier processes.” (Site C)
[FQHC] used [list methods]. Let us start with [method 1], what would have made that easier? What was hard about [method 1]? [Repeat for additional methods.]	• “[Administrative task] is a little bit more tricky to track how much time we’re actually putting into [task] because often, it’ll be over the course of several days waiting for an email to come back.” (Site B) • “…If there is a way that you can calculate the time from when the MA opens the chart to the time that a side provider opens the chart, … I think that’s the closest as you’re going to get in live documentation… But the problem with that is, …if at the end of the visit the provider does their piece but sends the MA … back in to do the teaching, I’m not really sure that time would be found in a measurable way …” (Site C)

Respondent roles: population health manager, nurse practitioner, navigator, and lab supervisor

## Results

### Participant roles

The FQHC staff involved in the bundled screening intervention could generally be classified into three categories: those responsible solely for implementation activities such as strategic planning and workflow design (chief medical officers, practice leads, advanced practice providers, and physicians); those in supervisory, quality improvement, and administrative roles related to delivering the intervention (population health managers, lab supervisors); and those whose roles involved direct interaction with patients (advanced practice providers, navigators).

### Passive data collection

Passive data from administrative sources on meetings devoted solely to implementing the bundled screening intervention were simple to collect and were available from all four sites, completely capturing the number and duration of meetings that took place. Total time for these meetings varied considerably (0.5–5.5 h per month). Additional meetings not *solely* devoted to the bundled screening intervention were noted; however, only one site (C) reported estimates of the time specifically devoted to bundled screening within these meetings (0.5 h per month). Each site reported that implementation activities also took place outside of formal meetings.

### Active data collection

Three of four sites (B, C, D) provided estimates from method 1a on the time they spent in the past week on bundled screening activities (record keeping, patient outreach, patient counseling). Two of the sites reported that competing priorities during that week made such estimates challenging (C) or impossible (A) to generate (Table 1).

When asked to estimate the time spent on bundled screening in a typical half-day clinical session (method 1b), one site (A) made an estimate and another (D) indicated it was “hard to say.” Respondents at three sites (A, B, C) reported the typical number of patients contacted about bundled screening rather than the specific amount of time spent on bundled screening activities during the session.

All personnel directly involved with bundled screening outreach at each of the four sites reported time use for completing the activity for “a typical patient” (method 2). Times were reported as intervals with ranges that included “2–10 min,” “5–15 min,” and “15–20 min.” Respondents noted a variety of factors affecting the time to complete bundled screening outreach for a patient, mostly involving characteristics important for equity such as language spoken, adverse SDOH, and issues related to poverty or mental health. They did not, however, attempt to quantify the extra time required to screen these patients.

The use of spreadsheets to track time use devoted to bundled screening (method 3) was feasible—all sites used spreadsheets for some time use tracking. Estimates were plausible with relatively low variability within tasks (e.g., completion of SDOH screening) and occasional outliers (e.g., for patients in need of extended education on FIT screening). The spreadsheets, adapted from pre-existing uses, were not designed to easily track longitudinal outreach such as when patients required multiple phone calls before they were reached.

### Focus group assessments

Though questions related to time use data collection methods were included in the focus groups conducted at all four sites, only two (B and C) responded about the collection of time use data (Table 2). Both indicated that when spreadsheets were already in use for care management, for example, registry-generated lists of patients to be contacted for screening, it was a simple matter to actively document time use there. Passive data collection through time-stamped electronic health record (EHR) logins/logouts was also suggested as an efficient means of time tracking, although not feasible in the current study. More challenging were tasks that might be interrupted, for example, administrative tasks that play out over days.

## Discussion

In the current study, we endeavored to generate practical data collection advice for economic evaluations of EBPs conducted in resource-constrained settings by embedding a qualitative assessment of time use data collection in a pilot study of a bundled screening intervention. With a dearth of existing guidance, we sought to identify the strengths and weaknesses of different data collection approaches through interactions with FQHC leadership and staff in order to inform equity-focused economic evaluations. At the same time, we wanted to assess different measures’ ease of use to better understand which methods would be least disruptive in resource-constrained settings.

Perhaps unsurprisingly, passive data collection was an appealing method of measuring time use. While these measures are simple and objective, the FQHC staff made clear that they were not always an accurate reflection of the time devoted to bundled screening. For example, time use measured from meeting records would overestimate time use when meetings covered topics beyond bundled screening. Conversely, not all implementation planning took place during formal, recorded meetings, so some relevant time use was missed. For other tasks, time-stamped EHR data were suggested as a promising strategy for passively collecting data on time use. However, times recorded in the EHR for screening-related efforts might underestimate time use if some of the work did not involve logging into and out of the EHR. Despite these imperfections, passive data collection may nevertheless provide an objective record of how *many* events took place. These data can then be supplemented by querying participants about time devoted to the EBP for each event. Careful process mapping is essential for understanding the extent to which passive data collection efforts may over- or underestimate the essential aspects of time use.

Three alternate approaches to actively self-reporting time use for bundled screening were compared. A question anchored by the phrase, “in the past week” (method 1a) was of limited utility because population health activities varied from week to week depending on shifting priorities. Anchoring the time use question on a “typical” session (method 1b) proved more tractable, though staff often reported the number of patients screened per session rather than the time it took to screen individual patients. Using spreadsheets or surveys (methods 2 and 3) to directly assess the time needed to screen a typical patient yielded usable numeric estimates from all four sites. Spreadsheet-based time-use measurement may be most successful when appended to existing processes that already employ spreadsheets and when equity-relevant patient characteristics can be linked to time-use estimates. Survey-based time-use measurement framed in terms of a “typical” event rather than a specific time period, and specifically seeking both the frequency and duration of the events, is also promising. Because the shortest and longest times reported by survey for a typical screening event differed up to five-fold, coupled with the range of patient characteristics driving longer screening times, subgroups relevant for a particular context should be identified prior to data collection, and time estimates should be generated for typical members of those subgroups. This is especially important for equity-focused economic evaluations.

Building data collection on time use into existing task-tracking systems (e.g., electronic health records and patient registries) was regarded as a promising method for objective data collection, acceptable to users, and holding high face validity for accuracy by not relying on self-report. Clearly, not all tasks are suited to this approach; such methods may be most applicable for population health management tasks.

Our emphasis on equity led us to focus on micro-costing techniques where the goal is to estimate the costs of individual tasks that together make up the EBP of interest [13]. Macro-costing, on the other hand, considers aggregate time use. Even in equity-focused economic evaluations, macro-costing may be used for time use components when they are reasonably believed to be unchanged with increased care delivery equity.

Our findings are subject to certain limitations. While strengths and weaknesses of different measures were identified, additional work is needed to validate time assessment methods against gold standards [14]. Data for our assessments were obtained from a small set of FQHCs and may not be representative of experiences at other FQHCs. Furthermore, assessments of time use data collection methods were a secondary aim of the focus groups, which may have limited some of the responses. Lastly, our assessments were conducted in the context of a pilot study during the first 2 years of the COVID-19 pandemic and need to be replicated in larger studies and varied contexts.

## Conclusions

Pragmatic methods to estimate the personnel time required for implementing new EBPs in FQHCs are necessary, whether for academic research or program planning. Our pilot work provides evidence directly from participating FQHCs on the strengths and weaknesses of different data collection approaches. Passively collected time use data are the least burdensome and should be pursued when possible. This data collection will need to be iterated and supplemented with qualitative assessments to ensure time use estimated from passive data sources is an accurate reflection of the effort devoted to an EBP. When workflows are already tracked by active data collection processes, these are also promising data collection methods. Self-reported time use will be most accurate when questions inquire about “typical” tasks and specific types of patients. Streamlining data collection methods and addressing important heterogeneity will ensure resource-constrained settings can gather accurate cost data for planning equity-focused activities.

## Data Availability

The data used and analyzed during the current study are available from the corresponding author upon reasonable request.

## Abbreviations

FQHCs: Federally qualified health centers
SDOH: Social determinants of health
CRC: Colorectal cancer
EBP: Evidence-based practice
EHR: Electronic health records

## References

[1] Aarons GA, Hurlburt M, Horwitz SM (2011). Advancing a conceptual model of evidence-based practice implementation in public service sectors. Adm Policy Ment Health.

[2] Damschroder LJ (2009). Fostering implementation of health services research findings into practice: a consolidated framework for advancing implementation science. Implement Sci.

[3] Glasgow RE (2019). RE-AIM planning and evaluation framework: adapting to new science and practice with a 20-year review. Front Public Health.

[4] Davlyatov G, Hiller S, Cendoma P, Borkowski N (2023). The self-sustainability of federally qualified health centers: examining the impact of Medicaid expansion. J Ambul Care Manage.

[5] Cidav Z (2020). A pragmatic method for costing implementation strategies using time-driven activity-based costing. Implement Sci.

[6] O’Leary MC (2022). Extending analytic methods for economic evaluation in implementation science. Implement Sci.

[7] Health Resources and Services Administration. Health Center Program Award Recipients. https://www.hrsa.gov/opa/eligibility-and-registration/health-centers/fqhc. Accessed 24 Oct 2022.

[8] Aschbrenner KA (2022). Stakeholder and equity data-driven implementation: a mixed methods pilot feasibility study. Prev Sci.

[9] Kruse GR (2023). Embedding community-engaged research principles in implementation science: the Implementation Science Center for Cancer Control Equity. J Clin Transl Sci.

[10] Kruse GR (2022). Implementing expanded COVID-19 testing in Massachusetts community health centers through community partnerships: protocol for an interrupted time series and stepped wedge study design. Contemp Clin Trials.

[11] Palinkas LA (2014). Qualitative and mixed methods in mental health services and implementation research. J Clin Child Adolesc Psychol.

[12] O’Brien BC, Harris IB, Beckman TJ, Reed DA, Cook DA (2014). Standards for reporting qualitative research: a synthesis of recommendations. Acad Med.

[13] Basu A. Estimating costs and valuations of non-health benefits in cost-effectiveness analysis. In: Neumann PJ, Sanders GD, Russell LB, Siegel JE, Ganiats TG, eds. Cost-effectiveness in Health and Medicine. 2nd ed. Oxford University Press. 2017. p. 218.

[14] Keel G, Savage C, Rafiq M, Mazzocato P (2017). Time-driven activity-based costing in health care: a systematic review of the literature. Health Policy.

